# Association of vitamin D receptor gene *BsmI* (A>G) and *FokI* (C>T) polymorphism in gestational diabetes among Saudi Women

**DOI:** 10.12669/pjms.316.7525

**Published:** 2015

**Authors:** Hesham A. El-Beshbishy, Manal A. Tawfeek, Inass M. Taha, Thoraya FadulElahi, Amal Y. Shaheen, Fouad A. Bardi, Intessar I. Sultan

**Affiliations:** 1 Prof. Hesham A. El-Beshbishy, Ph.D., M.Sc., Pharma B.Sc. Center for Genetics and Inherited Diseases and Medical Laboratories Technology Department, Taibah University, Madina, Saudi Arabia. Biochemistry Department, Faculty of Pharmacy, Al-Azhar University, Nasr City, Cairo 11751, Egypt; 2 Prof. Manal A. Tawfeek, Ph.D., MD. Biochemistry and Molecular Medicine Department, College of Medicine, Taibah University, Madina, Saudi Arabia Clinical Pathology Dept., Faculty of Medicine, Tanta University, Tanta, Egypt; 3 Dr. Inass M. Taha, MD., Internal Medicine Department, College of Medicine, Taibah University, Madina, Saudi Arabia; 4 Dr. Thoraya Fadulelahi, MRCOG., Department of Obstetrics and Gynecology, Ohud Hospital, Madina, Saudi Arabia; 5 Dr. Amal Y. Shaheen, SAB., Department of Obstetrics and Gynecology, Ohud Hospital, Madina, Saudi Arabia; 6 Fouad A. Bardi, Pharma B.Sc., King Fahd hospital, Madina, Saudi Arabia; 7 Prof. Intessar Sultan, M.D., Internal Medicine Department, College of Medicine, Cairo University, Egypt. Internal Medicine Department, College of Medicine, Taibah University, Madina, Saudi Arabia

**Keywords:** Gestational diabetes, Vitamin D receptor, Polymorphism, *BsmI*, *FokI*

## Abstract

**Objective:**

Vitamin D receptor (VDR) gene polymorphism have a role in diabetes mellitus pathogenesis. Present study was conducted to determine VDR gene variants among Saudi gestational diabetics (GDM) in Madina, KSA.

**Methods:**

This cross sectional study was conducted on 112 GDM patients and 218 normal healthy control. Age, body mass index and blood pressure levels were recorded. Serum triglycerides (mg/dl), total cholesterol, HDL-cholesterol, LDL-cholesterol, fasting blood glucose FBG and post-prandial blood glucose PPBG were estimated. Extracted DNA template was amplified by PCR reaction and genotyped for single nucleotide polymorphism of *BsmI* and *FokI* by restriction fragment length polymorphism-PCR (RFLP-PCR) analysis.

**Results:**

FBG and PPBG levels in GDM patients were significantly elevated by +48.6% and +50%, respectively (*P*=0.005). Serum triglycerides, total cholesterol and LDL-cholesterol (mg/dl) levels in GDM patients were elevated significantly by +40.5% (P=0.005), +16% (*P*=0.01) and +30.8% (*P*=0.005), respectively. Serum HDL-cholesterol (mg/dl) showed significant decline by -10.5%. *FokI* VDR genotypes showed association with PPBG (*P*=0.05) among GDM patients. The F*f*, FF and *ff* genotype percentage among GDM patients was 48.2%, 30.4% and 21.4%, respectively. *FokI* (F and *f*) and *BsmI* (B and b) alleles frequency showed no significant difference between GDM patients and control. Percentage *BsmI* and *FokI* total homozygous and heterozygous variants among GDM was 45.5% and 81.4%, respectively.

**Conclusion:**

VDR *BsmI* and *FokI* polymorphic marker not associated with Saudi GDM.

## INTRODUCTION

Vitamin D deficiency is associated with diabetes mellitus.^1^-^3^ Vitamin D receptor (VDR) gene variants may contribute to development of diabetes mellitus through calcium metabolism alteration and modulation of insulin secretion.^4^-^6^

VDR gene having 6 genes polymorphic forms. Among them; *FokI* restriction fragment length polymorphism (RFLP) in exon 2 and *Bsm1* RFLPs which located between exons 8 and 9. Polymorphism located at 3end of VDR gene *BsmI* (A to G), have unknown functional effects.^7^ Positive association between *BsmI* (genotype bb) polymorphisms with reduced insulin secretory capacity was reported.^8^ Diabetics with BB genotype of *BsmI* allele in VDR gene presented higher levels of C-peptide suggesting association between VDR polymorphism and diabetes.^9^

Gestational diabetes (GDM) patients are known to be at greater risk of reduced vitamin D levels as vitamin D was deficient in 70.6% of GDM patients.^10^,^11^ This study was carried out in order to investigate association between VDR-*BsmI*(A>G) and VDR-*FokI* (C>T) gene polymorphism and risk of GDM among Saudi pregnant women located in Madina-Saudi Arabia.

## METHODS

Blood samples were collected from 112 pregnant GDM and 218 normal healthy control Saudi women from outpatient clinic of Ohud hospital, Madina, Saudi Arabia from 11-2013 till 11-2014. Local ethical committee of Medical Applied Sciences faculty, Taibah University, Saudi Arabia approved study protocol and written informed consent was obtained from participants. Age (years), body mass index-BMI (Kg/m^2^), systolic blood pressure (mm/Hg) and diastolic blood pressure (mm/Hg) levels were recorded. Serum triglycerides (mg/dl), total cholesterol (mg/dl), HDL-cholesterol (mg/dl) and LDL-cholesterol (mg/dl) levels were estimated using kits from Randox, UK. Fasting blood glucose FBG (mg/dl), post-prandial blood glucose PPBG (mg/dl), were determined using glucose oxidase kit from Randox, UK. Patients screeningwas carried out according to American Diabetes Association guidelines.^12^ Exclusion criteria included diabetic women, women with multiple pregnancies or diseases as infections, nephropathy, retinopathy,…. etc, and smoking.

Five ml blood sample was collected in EDTA-tube. DNA was extracted using GelElute DNA extraction kit (QIAGEN, Germany) and was checked for purity and concentration.^13^ DNA samples were all quoted and stored at -20°C till analysis.

DNA (2 µl) was amplified in 50 µl PCR reaction mixture containing 5 µl10x PCR buffer, 25 mM MgCl_2_, 4 m MdNTPs, 15.M of each primer and 0.5U*Taq* DNA polymerase. Primers used in this study are listed in Table-I. PCR thermal cycler (SwiftMaxi thermal cycler, ESCO Technologies Inc., USA) was programmed as follows: denaturation step at 95°C for three minutes followed by 35 cycles as follows:- 94°C for 20 sec; 60°C for 40 seconds and 72°C for one minute. Final extension cycle at 72°C for 5 minutes was done. PCR reactions were set up separately for *FokI* and *BsmI* polymorphic sites of VDR gene. PCR products were analyzed on 2% agarose gel and UV-visualized.

**Table-I:** Primers used for polymerase chain reaction (PCR)of VDR *FokI* and *BsmI* genes.

Polymorphism	Primer
Exon 7/Intron 8 (A/G)	F 5`CAACCAAGACTACAAGTACCGCGTCAGTGA3`
Bsm1 rs1544410	R 5`AACCAGCGGGAAGAGGTCAAGGG 3`
Exon 2 (T/C)	F 5`AGCTGGCCCTGGCACTGACTCTGCTCT 3`
Fok1 rs2228570	R 5`ATGGAAACACCTTGCTTCTTCTCCCTC 3`

VDR: Vitamin D receptor.

Restriction fragment length polymorphism (RFLP-PCR) was used to identifyVDR genotypes. Amplified PCR product (10 ml) was digested (37°C for 20h) with 4U of either *BsmI* or *FokI* restriction enzyme (NEB, UK) in 20 µl reaction volume. Digested product was electrophoresed on 2% agarose gel. For statistical analysis, capital letters represented absence and lowercase letters represented presence of *BsmI* or *FokI* restriction site; (B/b) and (F/*f*), respectively. Genotype was determined according to fragments length i.e. homozygote GG(BB) subjects = 650 and 172bp product; heterozygote GA (Bb) subjects = 822, 650and 172bp products and homozygote AA (bb) subjects =822bp product. SNP resulting in A-G substitution in VDR gene intron 8 leads to generation of a *BsmI* restriction site. Homozygous subjects with alleles containing nucleotide A at this position showed one band at 822 bp and were designated as having bb *BsmI* genotype. Homozygous subjects with alleles containing G at this position showed 2 bands of 650 and 172bp and were designated as BB. Subjects with heterozygote status showed 3 bands:825, 650 and 172bp and were designated Bb.^14^

*FokI* genotype was determined according to fragments length i.e. homozygote (FF) subjects = 196, 69bp product;heterozygote (F*f*) subjects =265, 196 and 69bp products and homozygote (*ff*) subjects =265bp product. SNP resulting in T-C substitution in exon2 of VDR gene leads to the generation of a *FokI* restriction site. Homozygous subjects with alleles containing nucleotide T at this position showed an intact 265bp band and were designated as having *ff FokI* genotype. Homozygous subjects with alleles containing C at this position showed 2 bands of 196 and 69bp (FF Subjects). Heterozygote subjects showed all 3 bands: 265, 196 and 69bp and were designated F*f*.^15^

Results were presented as means±standard error (SE) and number (percentage). Difference between two groups was determined by unpaired Student`s t-test using GraphPad Prism software version 5.0 (USA). Results were considered statistically significant at P ≤0.05.

## RESULTS

Total number of 112 GDM patients and 218 healthy pregnant women were recruited in our study. Age, BMI, systolic and diastolic blood pressureshow no statistical significant difference compared to normal control (Table-II).

**Table-II:** Demographic and biochemical data of GDM patients and controls.

Parameter	GDM patients (n= 112)	Controls (n=218)	P value
Age (years) % change from control	41±4.1+2.4%	40±3.1	0.9
BMI (Kg/m^2^) % change from control	24.8±1.4 +8.5%	22.7±1.3	0.5
B.P. systolic (mm/Hg) % change from control	131±3.6 +7.6%	121±4.1	0.2
B.P. diastolic (mm/Hg) % change from control	82±3.6 +4.8%	78±3.6	0.6
FBG (mg/dl) % change from control	175±6.1 +48.6%	90±4.2	0.005
PPBG (mg/dl) % change from control	260±7.1 +50%	130±5.2	0.005
Triglycerides (mg/dl) % change from control	185±5.1 +40.5%	110±4.2	0.005
Total cholesterol (mg/dl) % change from control	176±4.8 +16%	148±5.6	0.01
HDL-cholesterol (mg/dl) % change from control	38±1.7 -10.5%	42±1.4	0.2
LDL-cholesterol (mg/dl) % change from control	146±5.2 +30.8%	101±4.3	0.005

Data were expressed as mean+SE. BMI: body mass index; HDL-C: high density lipoprotein-cholesterol; LDL-C: low density lipoprotein-cholesterol; B.P.: blood pressure; FBG: fasting blood glucose; PPBG: post-prandial blood glucose.

FBG and PPBG levels in GDM patients were significantly elevated by +94.4% and +50%, respectively, compared to normal control (*P*=0.005). Serum triglycerides, total cholesterol and LDL-cholesterol levels in GDM patients were elevated significantly by +68.2% (*P*=0.005), +18.2% (*P*=0.01) and +44.55% (*P*=0.005), respectively, compared with normal control. Whereas serum HDL-cholesterol showed non-significant decline by -9.1%, compared to normal control (*P*=0.2) (Table-II).

**Fig.1:**
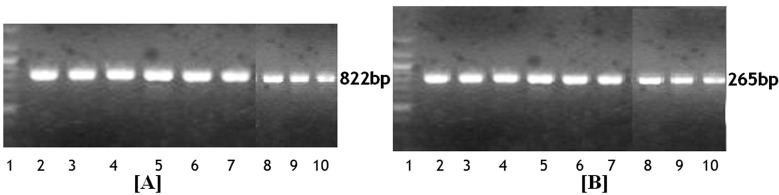
2% agarose gel electrophoresis of VDR PCR for [A] VDR SNPs BsmI enzyme showing a wild type bb 822bp product [B] VDR SNPs FokI enzyme showing a wild type ff 265bp product. Lane 1: 100bp DNA ladder, Lanes 2-10: PCR product DNA. PCR: polymerase chain reaction; SNPs: single nucleotide polymorphism; VDR: vitamin D receptor.

Fig.1, shows PCR product of 822 bp for VDR *BsmI* gene and 265 bp for VDR *FokI* gene. We compared *FokI* and *Bsm*I VDR genotypes with demographic and biochemical parameters of GDM patients but we could not achieve significant association of any of the parameters except PPBG (*P*=0.05) with VDR *FokI* genotypes (Table-III).

**Table-III:** Association of various biochemical and clinical parameters with different VDR (*BsmI* and *FokI*) genotypes in GDM.

Parameter	BsmI		P value	FokI		P value
	bb	BB + Bb		ff	FF+ Ff	
Age (years)	42±3.1	40±4.1	0.8	41±3.1	41±2.1	0.9
BMI (Kg/m^2^)	23.8±1.4	22.9±1.4	0.8	24.8±1.4	24.8±1.4	0.95
B.P. systolic(mm/Hg)	134±4.5	136±3.6	0.9	131.5±3.5	131.7±3.3	0.988
B.P. diastolic(mm/Hg)	81±3.4	86±3.2	0.5	81.3±3.6	81.5±3.6	0.98
FBG (mg/dl)	165±7.1	175±5.9	0.5	161.4±6.1	171.5±6.1	0.4
PPBG (mg/dl)	249±8.1	265±7.8	0.4	239±7.1	268±7.1	0.5
Triglycerides (mg/dl)	191±4.0	175±4.8	0.1	176±4.1	185±6.1	0.4
Cholesterol (mg/dl)	177±3.7	166±3.9	0.2	172±3.3	169±5.2	0.8
HDL-C (mg/dl)	37.4±1.6	37.15±1.9	0.95	37.6±1.7	36.9±1.6	0.9
LDL-C (mg/dl)	155±8.2	161±7.2	0.7	155.6±4.2	156±3.9	0.98

Data were expressed as mean+SE. BMI: body mass index; HDL-C: high density lipoprotein-cholesterol; LDL-C: low density lipoprotein-cholesterol; B.P.: blood pressure; FBG: fasting blood glucose; PPBG: post-prandial blood glucose. bb, ff: wild genotypes; BB, FF: homozygous variants; Bb, Ff: heterozygous variants.

Genotype and allele frequency of VDR *FokI* and *BsmI* gene polymorphism in GDM patients and controls showed insignificant differences (Table-IV). Table-V, shows that *BsmI* total percentage of homozygous and heterozygous variants among GDM patients and control was 45.5% and 81.4%, respectively. *FokI* total percentage of homozygous and heterozygous variants among GDM patients and control was 78.6% and 84.8%, respectively.

**Table-IV:** Genotypes and allele frequenciesof VDR BsmI and FokI gene polymorphism.

Genotype/Allele frequency			RFLP-PCR products (bp)	GDM, no (%) n= 112	Control, no (%) n= 218	P value
*BsmI Genotype frequency*						
Wild	AA bb		822	61 (54.5)	40 (18.3)	0.820
Homozygous variant	GG BB		650, 172	11 (9.8)	66 (30)	
Heterozygous variant	GA Bb		822, 650,172	40 (35.7)	112 (51.4)
BsmI Allele frequency						0.236
		Allele B		45 (40.2)	101 (46.3)	
		Allele b		67 (59.8)	117 (53.7)	
*FokI Genotype frequency*						
Wild	CC *ff*		265	24 (21.4)	33 (15.1)	0.341
Homozygous variant	TT F*f*		196, 69	34 (30.4)	65 (29.8)	
Heterozygous variant	TC F*f*		265,196, 69	54 (48.2)	120 (55)	
*FokI Allele frequency*						
		Allele F		40 (35.7)	123 (56.4)	0.100
		Allele *f*		72 (64.3)	95 (43.6)	

RFLP-PCR: restriction fragment length polymorphism; VDR: vitamin D receptor.

**Table-V:** VDR gene *BsmI* and *FokI* gene polymorphism (homozygous + heterozygous) variants of the study groups.

Genotype	GDM, no (%) n= 112	Control, no (%) n= 218
*BsmI*		
Wild type	61 (54.5)	40 (18.3)
Variant (homo and heterozygous)	51 (45.5)	178 (81.4)
*FokI*		
Wild type	24 (21.4)	33 (15.1)
Variant (homo and heterozygous)	88 (78.6)	185 (84.8)

VDR: vitamin D receptor.

## DISCUSSION

Diabetes mellitus has become major health concern worldwide. About 90-95% of the total diabetics are of type 2.^16^ Diabetes mellitus affect about 30% of Saudi population. Females <50 years old (gestational age) had greater prevalence than males.^17^ VDR polymorphism influences susceptibility to type 1 diabetes mellitus, but association with GDM is not yet clear.^18^ Vitamin D has suggested to be strongly related to pancreatic β-cell function and insulin sensitivity.^8^,^19^ It was reported that, no association was noticed between VDR *BsmI* polymorphism and GDM in Saudi population.^20^

We analyzed VDR gene *BsmI* (A>G) and *FokI* (C>T) polymorphic markers in GDM pregnant Saudi women. It was revealed that, VDR gene polymorphic markers were not found to be associated with VDR gene polymorphic markers with type 1 diabetes mellitus.^21^

Genotypes frequencies for wild, heterozygous and homozygous variant of *BsmI* polymorphic allele among control groups were 0.183, 0.300 and 0.514, respectively, and among GDM patients were 0.545, 0.0980 and 0.357, respectively, with insignificant association (*P*=0.820). Total homozygous and heterozygous was 0.455 and 0.814 among GDM patients and control, respectively (Table IV & V). It was reported that, *BsmI* genotype frequencies in GDM Saudi subjects were 0.300, 0.100 and 0.600, respectively; with no significance.^20^ Genotypes frequencies for wild, heterozygous and homozygous variant of *FokI* polymorphic allele in control were 0.151, 0.298 and 0.551, respectively, and in GDM patients were 0.214, 0.304 and 0.482, respectively, withno significance (*P*=0.341). Total homozygous and heterozygous was 0.786 and 0.848 among GDM patients and control, respectively (Table IV & V). This is the first time that, VDR gene *Fok1* polymorphic allele was analyzed in Saudi GDM subjects. Other studies have revealed that *Fok1* genotype frequencies among Indians were 0.437,0.491 and 0.07221.^22^ Both FBG and PPBG did not show significant different between those with wild type and variant allele of both *BsmI* and *FokI* gene polymorphism among control and GDM subjects (Table-VI). These findings, are in contrast with results of other studies that showed direct association between *BsmI* and elevated FBG.^15^ An Indian study suggested that, VDR gene polymorphism is associated with type 2 DM.^23^

We reported no evidence of allelic or genotypic association of the *BsmI* and *FokI* of VDR gene with GDM in our studied GDM population. Results obtained by different investigators who studied *BsmI* polymorphism varied among diabetics. *BsmI* polymorphism has been linked to susceptibility to diabetes in several countries.^5^,^24^ However, studies in other countries could not establish association between *BsmI* and existence of diabetes.^25^,^26^ In type 2 DM, a link between *BsmI* and the onset of the disease has been found in several countries.^27^,^28^ but not amongother populations.^29^,^30^ These variations may due to ethnic differences in VDR polymorphisms distribution that may have role in diabetes mellitus pathogenesis.

## CONCLUSION

Both *BsmI* (A>G) and *FokI* (C>T) VDR gene polymorphism showed non-significant association with GDM subjects except for PPBG in case of *FokI* genotype. Polymorphic marker alleles did not have effect on glycemic of Saudi GDM patients. Further merit investigation will be required to elucidate these findings using larger sample size.
